# Level of elderly-supportive infrastructure, fine particulate matter and cardiovascular disease hospitalisations: a time-stratified case-crossover study in Wuhan

**DOI:** 10.1080/16549716.2024.2447651

**Published:** 2025-01-17

**Authors:** Mengxue Qin, Xingyuan Liu, Luyao Wang, Tengchong Huang, Xiuran Zuo, Yuliang Zou

**Affiliations:** a Center of Health Management, School of Public Health, Wuhan University, Wuhan, China; b Statistics Department, Wuhan Health Information Center, Wuhan, China; c Wuhan Health Information Center, Wuhan, China

**Keywords:** Built environment, PM_2.5_, cardiovascular disease, mitigating effects, urbanisation

## Abstract

**Background:**

Amid rapid urbanisation, the health effects of the built-environment have been widely studied, while research on elderly-supportive infrastructure and its interaction with PM_2.5_ (PM, Particulate Matter) exposure remains limited.

**Objectives:**

To examine the effect of PM_2.5_ on cardiovascular hospitalisation risk among the elderly and the moderating role of elderly-supportive infrastructure in Wuhan, a city undergoing rapid urbanisation.

**Methods:**

A time-stratified case-crossover design was adopted in which the K-means cluster analysis was applied to categorize elderly-supportive infrastructure. The correlation of PM_2.5_ with cardiovascular hospitalisations and the moderating role of elderly-supportive infrastructure were elucidated through the conditional logistic regression and z-test. Nonlinear relationships among variables were determined using restricted cubic splines.

**Results:**

173,486 case days and 589,188 control days were included. The cumulative lag effect of PM_2.5_ increased over time, peaking at 5 days. For every 10 µg/m^3^ increase in PM_2.5_, the risk of hospitalisation rose by 1.5% (OR = 1.0150, 95% CI: 1.0113–1.0190). The aforementioned effect of PM_2.5_ exposure on health did not differ among varying levels of elderly-supportive infrastructure within a 300 m buffer zone. When the buffer zone was extended to 500 and 1000 m, a higher level of elderly-supportive infrastructure mitigated the adverse effects of short-term PM_2.5_ exposure on cardiovascular hospitalisations (*p* = 0.013), particularly for stroke (*p* = 0.017) and ischaemic heart disease (*p* = 0.026).

**Conclusions:**

Our findings suggest that high-level elderly-supportive infrastructure may protect against the adverse effects of PM_2.5_ on cardiovascular hospitalisation, highlighting the need to optimize elderly-supportive infrastructure for its health benefits in the elderly.

## Paper context

- **Main findings**: High level of elderly-supportive infrastructures may mitigate the adverse effects of short-term PM_2.5_ exposure on hospitalisations for cardiovascular disease, specifically for stroke and ischemic heart disease.
- **Added knowledge**: This is the first study to investigate the moderating effects of elderly-supportive infrastructure on short-term PM_2.5_ exposure and its impact on cardiovascular diseases, highlighting the potential benefits of promoting the cardiovascular health of the elderly through thoughtful planning and development of urban facilities.
- **Global health impact for policy and action**: Amid urbanisation and an aging population in many parts of the world, greater attention should be given to the design and planning of elderly-supportive infrastructure to mitigate the negative impacts of air pollution and enhance cardiovascular health outcomes for the elderly.

## Background

The world is experiencing rapid urbanisation. The Population Division of the United Nations Department of Economic and Social Affairs [1] has shown that most of the global population will migrate to urban regions by 2050. The total size of global cities may increase to 5.9 times by 2100 compared with the size in 2000 [2]. Compared with less densely populated regions, urban residents are greatly exposed to adverse environments [3], such as environmental noise, light pollution, severe air pollution, unreasonable building environments and insufficient green spaces.

Although urbanisation provides employment opportunities, high income, quality education and medical resources (which improve quality of life), the rapid increase in population density increases the demand for housing, public facilities and infrastructure. Urbanisation and changes in the built environment pose challenges to urban planning and management. Therefore, evidence-informed planning and efficient management are essential because of the intensified utilisation of land. For example, increasing green space and public areas, improving air quality and managing waste can foster healthy living environments for residents. Furthermore, an efficient public transportation system and sound urban planning can decrease travel time, alleviate traffic congestion and enhance overall convenience. An optimised urban design can enhance communication and social interaction, social cohesion and the well-being of residents [4–8].

The renewal and improvement of urban infrastructure are associated with the development of early CVD (cardiovascular diseases) and CVD-related risk factors [9–13]. For example, abundant green space [14] can improve cardiovascular function and reduce the risk of CVD. In contrast, exposure to high-density traffic is highly correlated with elevated levels of coronary artery calcification [15,16], particularly in males and individuals with low socio-economic status. However, a study conducted among African-American adults [17] did not find a significant association between residential proximity to traffic-related pollution and atherosclerosis, whether in the form of peripheral artery disease (PAD), coronary artery calcification (CAC) or abdominal aortic calcification (AAC). The built environment may interact with air pollution and health determinants, such as loneliness and social deprivation [18], influencing behavioural patterns through specific infrastructure, thereby impacting health, especially in the elderly. The level of community infrastructure is significantly associated with health outcomes, such as cognition [19], depression [20,21], well-being and satisfaction [22], dementia [23] and frailty in middle-aged and older adults. To date, most studies on the built environment have focused on single characteristics, such as proximity to roads [24], green spaces [25], distance to supermarkets [26,27], community retail environment [28], and accessibility to unhealthy food [29]. However, people in modern society experience a complex array of environmental exposures, including the built environment and air pollution, through their daily activities of living, working and traveling. Therefore, focusing on a single aspect of environmental exposure may underestimate the complexity of the environmental health effects.

The rapid progression of industrialisation and urbanisation in some regions has increased air pollution and related health problems [30,31]. One of the most concerning pollutants in ambient air is particulate matter (PM), particularly fine particles such as PM_2.5_. PM_2.5_ refers to a mixture of inhalable particles with an aerodynamic diameter of 2.5 µm or less in ambient air generated by various natural and human activities. PM_2.5_ can penetrate the alveoli through respiration, posing a threat to public health even at minimal exposure levels. The relationship between the built environment and air pollution [8] evolves with the development of socio-economic conditions due to rapid urbanisation. The results of a geographic Durbin model in the Yangtze River Delta region [32] estimated an inverted U-shaped relationship between urban expansion and PM_2.5_ pollution. The positive correlation may be reversed when the proportion of the urban built-up area reaches 0.21. Numerous epidemiological studies have demonstrated that short- and long-term exposures to PM_2.5_ are associated with an increase in incidence rate, hospitalisation and mortality of CVD [33–36]. A study conducted in South Korea [37] found that the risk of first hospitalisation for all CVD increases by 11.6% for every 2.9 μg/m^3^ increase in average annual PM_2.5_ exposure. Also, a time-stratified case-crossover study conducted in the United States [38] showed that the hospitalisation rate of CVD increases by 0.9% in adults aged 65 and older for every 2.9 μg/m^3^ increase in PM_2.5_, but it did not explore the impact of short-term PM_2.5_ exposure on hospitalisations for specific CVD, such as IHD (ischaemic heart disease) and stroke.

In the context of the aging populations worldwide, the wellbeing of elderly individuals who rely on community environments and facilities requires close attention. Older adults are highly susceptible to the surrounding environment due to decreased physical function and immunity. Additionally, the older adults are detached from their original social and work environments, experiencing shrinking social networks, reduced social interaction, and increased self-managed time compared with those under 60 years old. As a result, they may spend more time in the built environment and may be more sensitive to changes and perceptions [39]. Environmental factors, particularly walkability, entertainment facilities and green spaces can promote healthy aging, thus attracting much attention.

Therefore, building an elderly-supportive society and a liveable environment is crucial for the well-being of the elderly. China has transitioned into an aging society, thus implementing relevant policies to promote the transformation of urban construction and expediting the modernisation of urban infrastructures and public service facilities to accommodate the needs of the elderly. Based on the consideration of various factors such as the travel purpose [40], service level [41], and living needs of the elderly [42], and drawing on previous literature [43], five types of elderly-supportive infrastructure facilities were selected in this study, namely, elderly care facilities, medical and health facilities, cultural and educational facilities, residential and transportation facilities and open spaces for public activities.

Although high-level infrastructure may reduce the risk of CVD hospitalisations by promoting physical activities, it increases the exposure to PM_2.5_, thereby offsetting the benefits of physical activities. Most previous studies have examined the built environment in isolation, ignoring its comprehensive effects. Besides, the combined impact of short-term PM_2.5_ exposure and specific types of infrastructure, especially elderly-supportive infrastructure, on CVD hospitalisations is not fully understood. The combined health effects of the built environment and PM_2.5_ should be assessed to help in developing targeted intervention strategies and recommendations for reducing the risk of CVD hospitalisation.

Wuhan, as the largest city in central China, has undergone rapid population growth and large-scale population aggregation, making it a typical example of China’s urbanisation process. The urbanisation rate of Wuhan’s permanent population in 2017, 2018 and 2023 were 80.04%, 80.29% and 84.79%, respectively, according to data from the Wuhan Municipal Bureau of Statistics [44]. This study was conducted in Wuhan and included five types of elderly-supportive infrastructure facilities to examine their distribution and assess the moderating effect of different levels of specific infrastructure on the risk of CVD hospitalisation due to short-term PM_2.5_ exposure.

## Methods

### Study area and population

Wuhan is a major city in central China and an important industrial, scientific and educational hub, as well as a comprehensive transportation centre. The city had about 13.774 million permanent residents by the end of 2023. According to the Population and Employment Statistics Department of the National Bureau of Statistics of China [45] and the World Health Organization [46], the elderly in this study were defined as adults aged 60 years and over. Only permanent residents of Wuhan admitted for CVD between 6 January 2017 and 31 December 2018 were included. Hospitalisation data were obtained from the Wuhan Health Information Centre, and the hospitals considered were the 53 secondary and tertiary hospitals in Wuhan capable of diagnosing and treating CVD and providing inpatient services, where the admission was registered. The main diagnosis was conducted at the time of hospitalisation. CVD were grouped into three categories based on ICD-10 codes: IHD (I20–I25), stroke (I60–I69) and other forms of heart disease (I30–I52) [47].

### PM_2.5_, daily mean temperature and relative humidity

Exposure to PM_2.5_, daily mean temperature and daily mean relative humidity were determined based on the permanent residence address information of the participants. The 1 km daily near-surface PM_2.5_ dataset was obtained from China’s high-resolution and high-quality PM_2.5_ dataset (ChinaHighPM2.5) [48], which is part of China’s high-resolution and high-quality air pollution dataset (CHAP) series [49,50]. Daily mean temperature and relative humidity data were extracted from the ECMWF website [51], produced on behalf of the Copernicus Climate Change Service.

### Elderly-supportive infrastructure

In this study, the term ‘elderly-supportive infrastructure’ was defined as the surrounding infrastructure facilities that may support the daily activities and health-related goals of the elderly. The five types of infrastructure facilities included elderly care facilities (elderly day care centres and home-based elderly care service centres), medical and health facilities (hospitals, clinics, health service centres, physical examination centres and pharmacies), cultural and educational facilities (elderly universities, elderly schools and elderly activity centres), residential and transportation facilities (subway and bus stations, supermarkets and farmers’ markets) and open spaces for public activities (urban parks, street green spaces and citizen squares).

The types and quantities of Points of Interest (POIs) were obtained using map coordinates from various platforms such as Baidu, Gaode and Tencent to identify elderly-supportive infrastructure. POI represent various geographical entities, such as healthcare, shopping, education and transportation facilities, by abstracting them into points. The points can then be integrated with technologies, such as Geographic Information System (GIS) and Global Positioning System (GPS), enabling the rapid and efficient reflection of the spatial distribution and structure of specific elements. The infrastructure facilities were categorised into three service radii (300, 500 and 1000 m) based on the classification of living circles and the service radii of various infrastructure types outlined in the Standard for Urban Residential Area Planning and Design (GB50180–2018) and the Code for Planning of City and Town Facilities for the Aged (GB50437–2007), which were implemented in 2018. The distribution of elderly-supportive infrastructure within these radii was then evaluated.

### Statistical analysis

In this study, a time-stratified case-crossover design was used at the individual level. Case day was defined as the date of admission, while the control days were the same days of the weeks in the same month and year as the admission date. This approach helps mitigate bias caused by individual differences and uncontrollable factors.

Continuous variables were expressed as mean ± standard deviations, while categorical variables were expressed as frequencies. Group comparisons were conducted using t-tests or chi-square tests. The elderly-supportive infrastructure was stratified into high, middle and low levels via K-means clustering. The effects of PM_2.5_ exposure on hospitalisations for total CVD, including IHD, stroke and other forms of heart diseases were assessed via conditional logistic regression. The moderating effect of elderly-supportive infrastructure on the relationship between PM_2.5_ exposure and CVD hospitalisation risk was examined by fitting separate models. A two-sample z-test was used to compare stratum-specific effect estimates.

$$Z=\frac{\beta_{2}-\beta_{1}}{\sqrt{SE_{2}^{2}+SE_{1}^{2}}}$$

Nonlinear relationship between PM_2.5_ exposure and CVD hospitalisation risk at different levels of elderly-supportive infrastructure was assessed via restricted cubic splines.

Data were processed and analysed using R 4.2.1. A two-sided *p* < 0.05 was considered statistically significant.

## Results

The five types of elderly-supportive infrastructure facilities across different buffer zones are shown in Table 1. A total of 388,435 POIs of elderly-supportive infrastructure were identified, of which residential and transportation facilities accounted for the majority, followed by medical and health facilities, cultural and educational facilities, open spaces for public activities and elderly care facilities. Most participants in all buffer zones (300, 500 and 1000 m) lived in areas with low- or middle-level elderly-supportive infrastructure, with only a few residing in high-level elderly-supportive infrastructure areas.

**Table 1.** Distribution of elderly-supportive infrastructure in different buffer zones.

		Total	Low	Middle	High
Buffer 300 m	Type I	0(1)	0(1)	0(2)	1(1)
	Type II	9(16)	8(16)	14(21)	20(12)
	Type III	6(10)	5(9)	12(17)	29(8)
	Type IV	143(353)	107(249)	763(340)	1911(262)
	Type V	0(1)	0(0)	2(15)	9(8)
Buffer 500 m	Type I	1(2)	0(1)	2(12)	3(1)
	Type II	26(40)	19(34)	51(33)	41(13)
	Type III	18(28)	14(23)	47(35)	83(38)
	Type IV	490(827)	304(580)	1514(728)	3467(583)
	Type V	1(2)	0(1)	4(9)	36(11)
Buffer 1000 m	Type I	3(6)	1(4)	7(6)	14(5)
	Type II	97(127)	50(84)	161(74)	209(91)
	Type III	67(102)	34(63)	144(102)	314(126)
	Type IV	1926(2884)	965(1678)	4076(1480)	9092(860)
	Type V	3(8)	3(3)	9(15)	71(65)

(1) Type I: elderly care facilities; (2) Type II: medical and health facilities; (3) Type III: cultural and educational facilities; (4) Type IV: residential and transportation facilities; and (5) Type V: open spaces for public activities.

The socio-demographic characteristics of participants with low, middle and high levels of elderly-supportive infrastructure in different buffer zones are shown in Table 2. A total of 173,486 participants (90,154 males and 83,332 females) aged over 60 years (mean age: 74.42) were included in this study. The average length of hospital stay was 9.84 days. Most participants (82,569) were diagnosed with IHD, followed by stroke (55,870) and other forms of heart disease (35,083). In addition, gender, marital status and length of hospital stay were different among the groups. Notably, participants with low-level elderly-supportive infrastructure were more likely to be younger, female, married and have shorter hospital stays. The proportion of IHD was significantly higher in the high-level elderly-supportive infrastructure group than in the low-level elderly-supportive infrastructure group.

**Table 2.** Sociodemographic characteristics of participants.

			Low		Middle	High	F/χ2	*P*
Buffer 300 m	Age		74.28 ± 8.49	75.37 ± 9.25		78.07 ± 8.53	183.94	<0.001
	Days of hospitalisation		9.75 ± 7.41	10.66 ± 8.71		14.28 ± 9.83	206.9	<0.001
	Sex	Male	79364(51.9)	10535(52.7)		255(53.6)	6.022	0.05
		Female	73672(48.1)	9439(47.3)		221(46.4)		
	Marital status	Unmarried	2064(1.3)a	191(1)b		4(0.8)ab		<0.001
		Married	130404(85.2)a	16711(83.7)b		310(65.1)c		
		Other	20541(13.4)a	3059(15.3)b		162(34)c		
	CVD type	Other forms of heart disease	31180(20.4)a	3819(19.1)b		84(17.6)ab	204.618	<0.001
		IHD	71933(47)a	10346(51.8)b		290(60.9)c		
		Stroke	49956(32.6)a	5812(29.1)b		102(21.4)		
Buffer 500 m	Age		74.15 ± 8.42	75.40 ± 9.10		78.59 ± 9.74	404.02	<0.001
	Days of hospitalisation		9.67 ± 7.30	10.59 ± 8.53		13.02 ± 9.72	286.34	<0.001
	Sex	Male	71576(51.8)a	18079(52.7)b		499(54.1)ab	10.676	0.005
		Female	66669(48.2)a	16239(47.3)b		424(45.9)ab		
	Marital status	Unmarried	1925(1.4)a	329(1)b		5(0.5)ab	311.088	<0.001
		Married	117903(85.03)a	28884(84.2)b		638(69.1)c		
		Other	18386(13.3)a	5096(14.9)b		280(30.3)c		
	CVD type	Other forms of heart disease	28372(20.5)a	(6563(19.1)b		148(16)c	278.739	<0.001
		IHD	64450(46.6)a	17583(51.2)b		536(58.1)c		
		Stroke	45451(32.9)a	10180(29.7)b		239(25.9)c		
Buffer 1000 m	Age		73.91 ± 8.28	75.26 ± 9.00		76.64 ± 9.62	599.44	<0.001
	Days of hospitalisation		9.45 ± 7.05	10.60 ± 8.42		10.94 ± 8.51	472.6	<0.001
	Sex	Male	57119(51.1)a	31286(53.7)b		1749(50.0)a	103.34	<0.001
		Female	54575(48.9)a	27011(46.3)b		1746(50.0)a		
	Marital status	Unmarried	1588(1.4)a	651(1.1)b		20(0.6)c	338.563	<0.001
		Married	94928(85)a	49849(85.5)b		2648(75.8)c		
		Other	15149(13.6)a	7786(13.4)a		827(23.7)b		
	Disease type	Other forms of heart disease	23066(20.6)a	11309(19.4)b		708(20.3)ab	491.016	<0.001
		IHD	51012(45.7)a	29784(51.1)b		1773(50.7)b		
		Stroke	37639(33.7)a	17217(29.5)b		1014(29)b		

(1) CVD: Cardiovascular disease; (2) a, b, c: same subscript letters indicate no difference in the proportion of category columns; different subscript letters indicate a significant difference in the proportion of category columns.

PM_2.5_ exposure results across different buffer zones and levels of elderly-supportive infrastructure are shown in Table 3. PM_2.5_ exposure was significantly different among the different levels of elderly-supportive infrastructure within the 300 and 500 m buffer zones. Besides, participants in the middle-level elderly-supportive infrastructure group had lower PM_2.5_ exposure than those in the low-level infrastructure group. However, PM_2.5_ exposure was not significantly different among the different levels of elderly-supportive infrastructure within the 1000 m buffer zone.

**Table 3.** PM_2.5_ exposure in different buffer zones.

		low	middle	high	F	*P*
Buffer 300 m	PM_2.5__lag0	48.65 ± 29.36a	47.78 ± 29.76b	48.89 ± 31.84ab	7.774	<0.001
	PM_2.5__lag01	48.65 ± 29.26a	48.12 ± 30.31b	49.44 ± 31.88ab	3.156	0.043
	PM_2.5__lag02	48.73 ± 29.74a	48.05 ± 30.38b	50.67 ± 32.43ab	5.783	0.003
	PM_2.5__lag03	48.76 ± 30.09a	47.96 ± 30.83b	49.68 ± 30.47ab	6.579	0.001
	PM_2.5__lag04	48.48 ± 29.59a	47.82 ± 30.73b	49.92 ± 30.88ab	4.966	0.007
	PM_2.5__lag05	48.47 ± 29.62	48.11 ± 30.80b	50.57 ± 33.51ab	2.552	0.078
	PM_2.5__lag1	48.65 ± 27.74a	47.95 ± 28.26b	49.17 ± 30.10ab	5.773	0.003
	PM_2.5__lag2	48.68 ± 26.66a	47.98 ± 27.01b	49.67 ± 28.70ab	6.427	0.002
	PM_2.5__lag3	48.70 ± 25.90a	47.98 ± 26.16b	49.67 ± 27.30ab	7.306	0.001
	PM_2.5__lag4	48.66 ± 25.21a	47.95 ± 25.46b	49.72 ± 26.53ab	7.526	0.001
	PM_2.5__lag5	48.63 ± 24.68a	47.97 ± 24.90b	49.86 ± 26.11ab	6.866	0.001
Buffer 500 m	PM_2.5__lag0	48.64 ± 29.37a	48.16 ± 29.55b	49.98 ± 30.61ab	4.846	0.008
	PM_2.5__lag01	48.65 ± 29.26ab	48.31 ± 29.84a	50.31 ± 31.05b	3.419	0.033
	PM_2.5__lag02	48.71 ± 29.70a	48.36 ± 30.22b	51.65 ± 32.67c	6.571	0.001
	PM_2.5__lag03	48.72 ± 30.03a	48.42 ± 30.72a	51.15 ± 32.02b	4.507	0.011
	PM_2.5__lag04	48.47 ± 29.55a	48.11 ± 30.38b	50.58 ± 31.43c	4.468	0.011
	PM_2.5__lag05	48.46 ± 29.57a	48.24 ± 30.45a	51.36 ± 33.23b	5.212	0.005
	PM_2.5__lag1	48.65 ± 27.76a	48.24 ± 27.97b	50.15 ± 28.97a	4.513	0.011
	PM_2.5__lag2	48.67 ± 26.67a	48.28 ± 26.81b	50.65 ± 28.00c	5.689	0.003
	PM_2.5__lag3	48.68 ± 25.91a	48.31 ± 26.04b	50.77 ± 26.96c	5.993	0.002
	PM_2.5__lag4	48.64 ± 25.21a	48.27 ± 25.37b	50.73 ± 26.28c	6.3	0.002
	PM_2.5__lag5	48.61 ± 24.67a	48.27 ± 24.81b	50.84 ± 25.91c	6.609	0.001
Buffer 1000 m	PM_2.5__lag0	48.49 ± 29.14	48.73 ± 29.91a	47.64 ± 29.74b	2.982	0.051
	PM_2.5__lag01	48.52 ± 29.10	48.75 ± 29.84	48.27 ± 30.81	1.329	0.265
	PM_2.5__lag02	48.60 ± 29.59	48.75 ± 30.16	48.93 ± 31.68	0.607	0.545
	PM_2.5__lag03	48.64 ± 29.95	48.73 ± 30.54	48.76 ± 31.16	0.195	0.822
	PM_2.5__lag04	48.41 ± 29.45	48.41 ± 30.13	48.68 ± 31.48	0.144	0.866
	PM_2.5__lag05	48.41 ± 29.49	48.45 ± 30.15	49.02 ± 32.12	0.728	0.483
	PM_2.5__lag1	48.51 ± 27.59	48.74 ± 28.20	47.96 ± 28.42	2.206	0.11
	PM_2.5__lag2	48.54 ± 26.54	48.74 ± 26.99	48.28 ± 27.34	1.367	0.255
	PM_2.5__lag3	48.56 ± 25.80	48.74 ± 26.17	48.40 ± 26.31	1.004	0.366
	PM_2.5__lag4	48.53 ± 25.13	48.67 ± 25.43	48.46 ± 25.69	0.635	0.53
	PM_2.5__lag5	48.51 ± 24.62	48.64 ± 24.85	48.55 ± 25.21	0.488	0.614

a, b, c: same subscript letters indicate no difference in the proportion of category columns; different subscript letters indicate a significant difference in the proportion of category columns.

Conditional logistic regression analysis was conducted to assess the effects of PM_2.5_ exposure on the risk of CVD hospitalisation in the elderly with different lag days (Figure 1a). Results showed that PM_2.5_ exposure on the day of admission had the greatest single-lag impact on the risk of CVD hospitalisation. The total CVD hospitalisation risk increased by 1% (OR = 1.010 and 95%CI: 1.0072–1.0120) for every 10 µg/m^3^ increase in PM_2.5_ exposure. The risk of hospitalisation for other forms of heart disease, IHD and stroke increased by 1.3% (OR = 1.013 and 95%CI: 1.0070–1.0180), 0.9% (OR = 1.009 and 95%CI: 1.0053–1.0120) and 0.92% (OR = 1.0092 and 95%CI: 1.0047–1.0140), respectively. Longer lag phase indicates a smaller risk, which may reflect the delayed biological response to PM2.5 exposure. Herein, the cumulative lag effect of PM_2.5_ increased over time, peaking at a lag of 5 days. The risk of total CVD, other forms of heart disease, IHD and stroke increased by 1.5% (OR = 1.0150 and 95%CI: 1.0113–1.0190), 1.5% (OR = 1.0150 and 95%CI: 1.0061–1.0230), 1.6% (OR = 1.0160 and 95%CI: 1.0103–1.0210) and 1.5% (OR = 1.0150 and 95%CI: 1.0079–1.0220), respectively, for every 10 µg/m^3^ increase in PM_2.5_ exposure.

**Figure 1.**
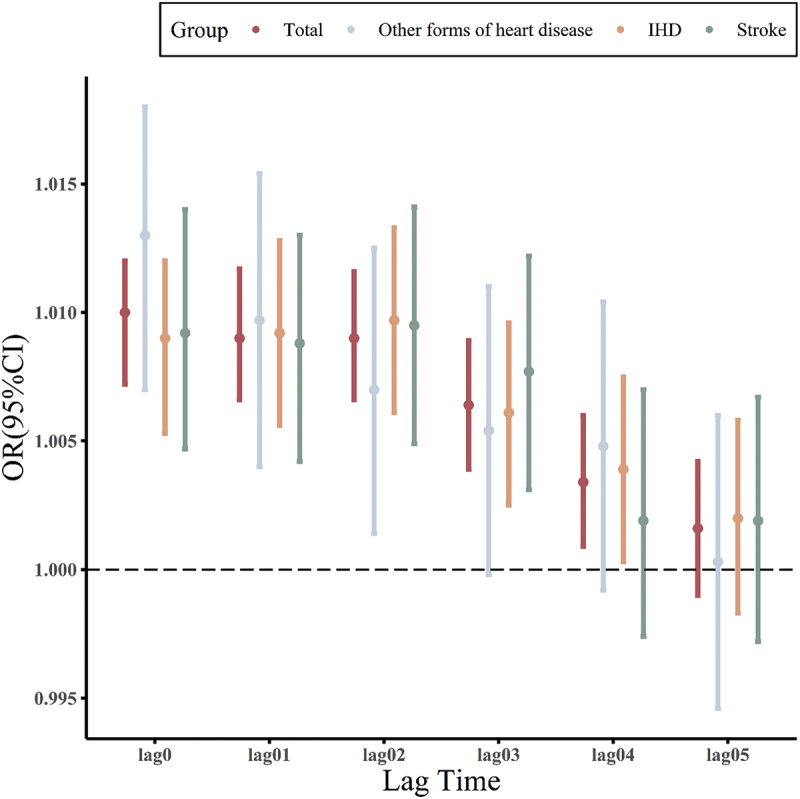
(a) Associations of PM_2.5_ exposure with CVD hospitalization on different single lag days. (b) Associations of PM_2.5_ exposure with CVD hospitalization on different cumulative lag days.

**Figure 1.**
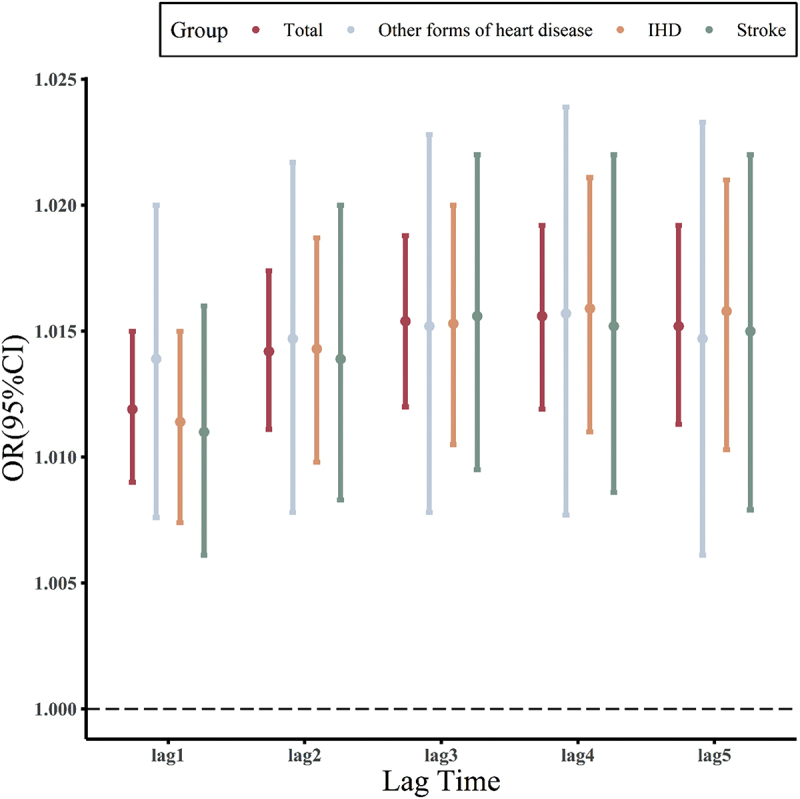
(Continued).

A stratified analysis was conducted based on the level of elderly-supportive infrastructure to further investigate the modification of different infrastructure levels. The results of the conditional logistic regression are shown in Table 4. The impact of PM_2.5_ on hospitalisation risk was not significantly different across various levels of elderly-supportive infrastructure in the 300 m buffer zone. In contrast, the impact of PM_2.5_ on hospitalisation risk for CVD (*p* = 0.013) and stroke (*p* = 0.017) was significantly different across the levels of elderly-supportive infrastructure within the 500 m buffer zone. Furthermore, an increase in PM_2.5_ exposure in the middle-level elderly-supportive infrastructure group did not statistically increase the hospitalisation risk for CVD (OR = 1.0055 and 95 CI:0.9969–1.0142) and stroke (OR = 0.994 and 95 CI:0.981–1.013). Similar results were found for IHD (OR = 1.0079 and 95 CI:0.999–1.017) within the 1000 m buffer zone. Notably, the risk of hospitalisation for other forms of heart disease caused by PM_2.5_ exposure was higher in the high-level infrastructure group than in the low-level infrastructure group. Sensitivity analysis, where the middle-level and high-level groups were combined, showed similar results. Also, the hospitalisation risk for other forms of heart disease caused by PM_2.5_ exposure was not significantly different between the low and middle levels of elderly-supportive infrastructure (Table A4). Separate regression analyses on the five types of specified infrastructure (Tables A1–A3) revealed no significant differences, except for type I (elderly care facilities) infrastructure within the 1000 m buffer zone (*p* = 0.026).

**Table 4.** Conditional logistic regression results of PM_2.5_ and risk of CVD hospitalisation in the elderly in different buffer zones.

Infrastructure levels		CVD				Other forms of heart disease				IHD				stroke			
		OR	95%CI		P_Ztest_	OR	95%CI		P_Ztest_	OR	95%CI		P_Ztest_	OR	95%CI		P_Ztest_
300 m	Low	1.016	1.0116	1.02		1.014	1.004	1.023		1.016	1.01	1.022		1.017	1.009	1.024	
	Middle	1.011	1	1.023	0.467	1.027	1.002	1.054	0.3212	1.012	0.997	1.028	0.635	1	0.979	1.021	0.136
	High	1.019	0.9443	1.099	0.938	1.152	0.712	1.058	0.1263	1.033	0.934	1.142	0.756	1.103	0.949	1.283	0.288
500 m	Low	1.018	1.0135	1.022		1.015	1.006	1.025		1.019	1.012	1.025		1.019	1.011	1.027	
	Middle	**1.006**	**0.9969**	**1.014**	**0.013**	1.013	0.994	1.033	0.8328	1.008	0.996	1.02	**0.119**	**0.997**	**0.981**	**1.013**	**0.017**
	High	1	0.9476	1.055	0.512	0.955	0.831	1.098	0.3882	0.974	0.907	1.047	0.23	1.076	0.974	1.19	0.282
1000 m	Low	1.017	1.0122	1.022		1.009	0.998	1.02		1.021	1.014	1.028		1.017	1.008	1.025	
	Middle	1.012	1.0049	1.018	0.19	1.021	1.006	1.036	0.1974	**1.0079**	**0.999**	**1.017**	**0.026**	1.012	0.999	1.024	0.546
	High	1.022	0.9944	1.051	0.731	**1.095**	**1.032**	**1.161**	**0.0074**	0.997	0.958	1.037	0.239	1.014	0.964	1.066	0.918

Bold values indicate *P*<0.05.

**Figure 2.**
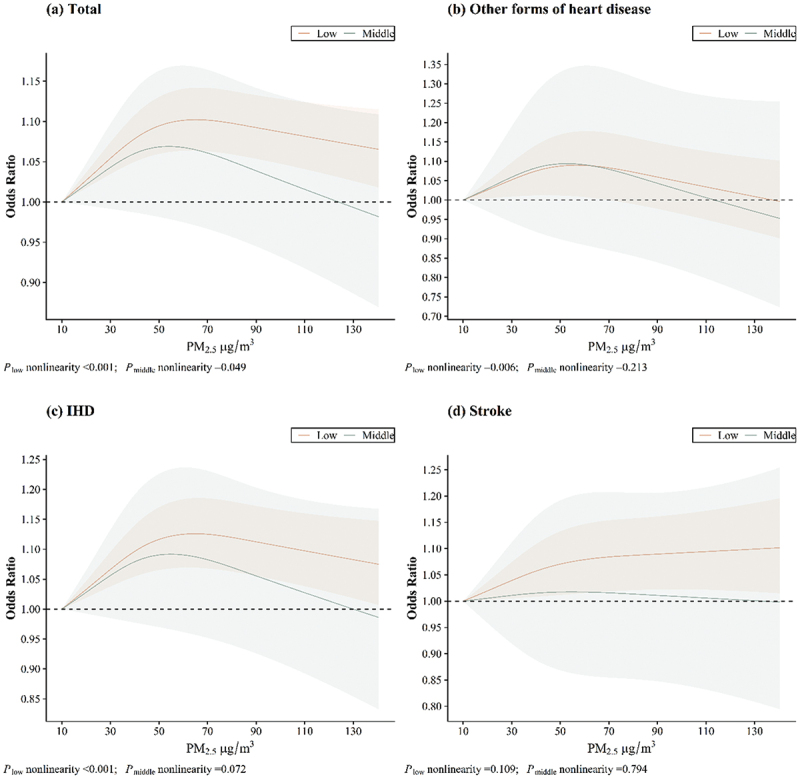
Exposure – response relationships between PM_2.5_ and CVD hospitalisation in 300 m buffer zone.

**Figure 3.**
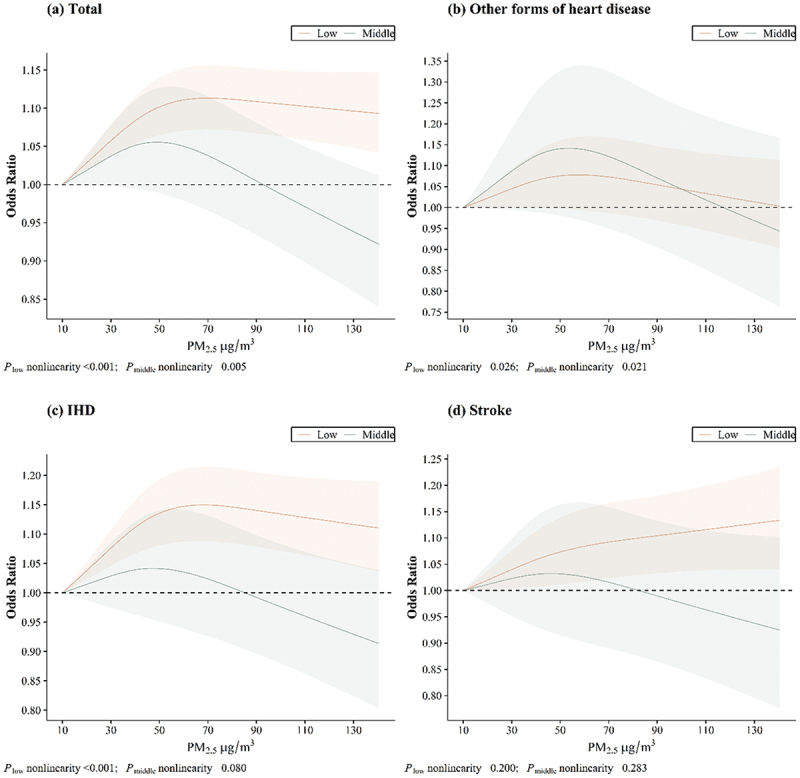
Exposure – response relationships between PM_2.5_ and CVD hospitalisation in 500 m buffer zone.

**Figure 4.**
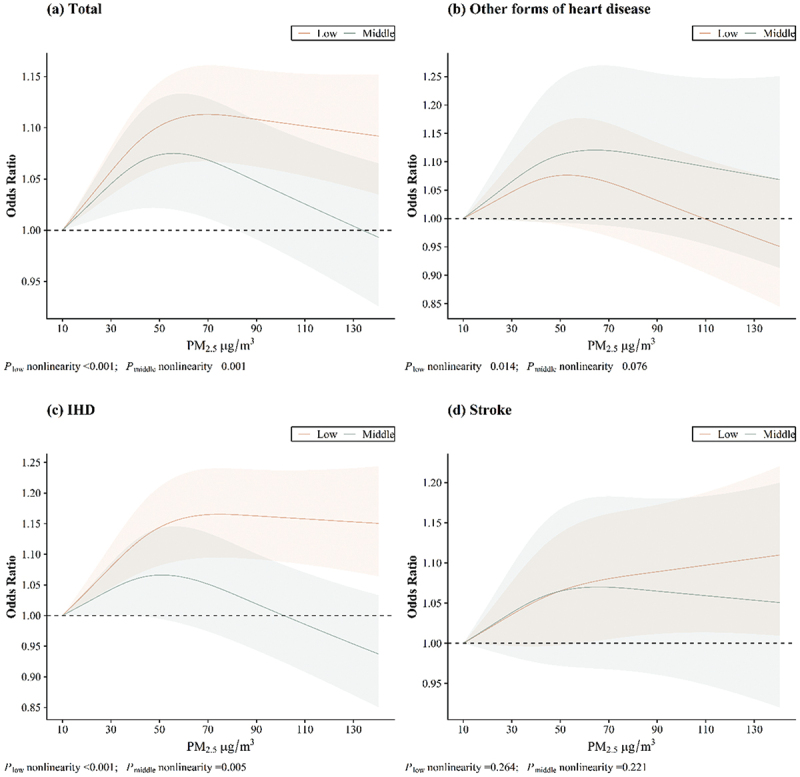
Exposure – response relationships between PM_2.5_ and CVD hospitalisation in 1000 m buffer zone.

The exposure – response relationships between PM_2.5_ exposure and CVD hospitalisation risk across different buffer zones are shown in Figures 2–4. The relationships between PM_2.5_ exposure and CVD hospitalisation risk were nonlinear, with a protective effect observed in areas with high levels of elderly-supportive infrastructure. Similar results were found between PM_2.5_ exposure and IHD hospitalisation risk (in the 1000 m buffer zone), but no saturation effect was observed. However, no nonlinear relationship was found between PM_2.5_ exposure and hospitalisation risk for stroke across any buffer zone.

## Discussion

To the best of our knowledge, this is the first study to investigate the distribution of elderly-supportive infrastructure and its moderating effects on the relationship between short-term PM_2.5_ exposure and CVD hospitalisation risk among the elderly. Most participants resided in community environments with low or middle levels of elderly-supportive infrastructure. The participants in areas with middle- and high-level infrastructure experienced lower PM_2.5_ exposure. The impact of PM_2.5_ exposure on hospitalisation risk for CVD gradually decreased, with increasing lag days. Notably, the cumulative effect increased over time. Furthermore, results showed that a high level of elderly-supportive infrastructure may mitigate the adverse health effects of short-term PM_2.5_ exposure, thereby benefiting cardiovascular health in the elderly.

Furthermore, short-term PM_2.5_ exposure was identified as a significant risk factor for CVD hospitalisations, consistent with previous studies [52]. A pooled analysis [53] found that a 10 μg/m^3^ increase in PM_2.5_ is positively associated with an increased risk of CVD incidence (RR = 1.12 and 95% CI = 1.05–1.19). In this study, the risks of hospitalisation for CVD, other forms of heart disease, IHD and stroke increased by 1.52%, 1.47%, 1.58% and 1.50%, respectively, for every 10 µg/m^3^ increase in PM_2.5_ exposure. This could be because the impact of PM_2.5_ exposure on cardiovascular health may be more pronounced in the elderly than in the younger populations. PM_2.5_ exposure impacts CVD through intracellular oxidative stress, mutagenicity/genotoxicity and inflammatory responses [54]. Endothelial dysfunction [55] and inflammation [56–58] play important roles in pathological and physiological processes. In addition, PM_2.5_ can induce cardiac tissue remodelling and functional changes by directly acting on the heart, leading to the occurrence and development of heart disease. The elderly have lower immune levels, poorer cardiopulmonary function, and are more exposed to air pollution, making them more vulnerable to the adverse effects of PM_2.5_ exposure.

In this study, results showed that a higher level of elderly-supportive infrastructure in the 500 and 1000 m buffer zones could mitigate the negative health effects of short-term exposure to PM_2.5_, positively influencing the cardiovascular health of the elderly. The elderly-supportive infrastructure may improve access to medical resources [59], exercise opportunities [60] and social connections [61,62], thus promoting health of older adults by increasing physical activity, reducing sedentary behaviour [63] and fostering social engagement. Notably, the limited area of the 300 m buffer zone likely contributed to the absence of significant differences among specific infrastructure, such as open space and green areas. This may also explain the lack of substantial variations in hospitalisation risk across different levels of elderly-supportive infrastructure within the 300 m buffer zone. Although no research has specifically focused on elderly-supportive infrastructure, some previous studies on specific infrastructure can support these findings [64]. For instance, Mason et al. found that the density and accessibility of facilities (gyms, parks and fast-food restaurants) [65] can greatly stimulate physical activity among the elderly, thereby reducing BMI. A study conducted in Japan [66] also found that being too far from sports facilities can restrict participation in physical activities. Studies conducted in Suzhou [67] and Shanghai [68] in China also indicated that social infrastructure can promote physical health of the elderly by influencing their total physical activity and duration. Furthermore, a study conducted in Hong Kong [69] suggested that a high-level infrastructure can improve the health of frail elderly aged over 60 years by promoting service utilisation, consistent with this study. Separate regression analyses indicate that elderly-supportive infrastructure may not operate independently but rather interactively to mitigate the adverse effects of PM_2.5_ on cardiovascular health in the elderly. Nonetheless, further studies should validate the impact of elderly-supportive infrastructure on cardiovascular health in older adults and elucidate the specific mechanisms.

Interestingly, the effects of elderly-supportive infrastructure on different subtypes of CVD were not uniform. Specifically, higher levels of elderly-supportive infrastructure were associated with lower hospitalisation risks for CVD, IHD and stroke in the 500 and 1000 m buffer zones. However, no evidence suggested that such infrastructure could moderate the hospitalisation risk for other forms of heart disease, particularly after reclassification (Table A5). This result may be due to the diverse pathogenesis of other forms of heart disease, such as pericarditis, endocarditis, heart valve disease and cardiomyopathy. Each subtype of other forms of heart diseases has distinct pathophysiological mechanisms and influencing factors [70–73], making the relationship between elderly-supportive infrastructure and hospitalisation risk complex. Therefore, future studies should focus on a more detailed CVD classification to thoroughly assess the impact of elderly-supportive infrastructure.

This study has several unique strengths. First, this was a time-stratified case-crossover study conducted at the individual level, allowing for the control of stable confounding factors, such as age and lifestyles. Second, unlike previous studies, five types of elderly-supportive infrastructure were examined in this study, considering the overall infrastructure around the place of residence of the elderly and the complexity of their community environment. Third, CVD was classified as accurately as possible to reflect the effects of PM_2.5_ exposure. Finally, this study was conducted in Wuhan, a rapid urbanising city, making it a representative setting for studying the health effects at the infrastructure level. Therefore, these findings may apply to other cities undergoing rapid urbanisation.

However, this study has some limitations. First, residential PM_2.5_ concentrations were used as exposure for elderly individuals instead of dynamic data. As a result, the measurements may deviate from the actual exposure conditions. However, the elderly population is more dependent on their surrounding environment, with a relatively fixed activity range, indicating that the error may be small. Meta-analyses have shown that considering spatial variability and the outdoor micro-environment does not significantly affect the effectiveness of using outdoor PM_2.5_ concentrations as an individual exposure profile [74]. Static exposure assessments using residential areas can provide accurate values. Second, CVD events were classified into different categories, including other forms of heart disease, IHD and stroke, which may mask the different effects of PM_2.5_ exposure and elderly-supportive infrastructure on the mechanisms of CVD. Also, a detailed classification of stroke was not achieved due to sample size constraints. Ischaemic stroke and haemorrhagic stroke have different pathogenesis, indicating varying effects of the built environment and PM_2.5_ exposure. Third, inherent correlations between infrastructure facilities cannot be eliminated. However, the correlation between 0.04 and 0.81 remains acceptable for highly correlated neighbourhood-built-environment variables [69]. Fourth, the population distribution across various regions is uneven. Besides, most hospitalised elderly participants came from areas with inadequate elderly-supportive infrastructure, which may lead to an underappreciation of the importance of such infrastructure. The low number of hospitalisations among the elderly in areas with high-level elderly-supportive infrastructure confirmed the health benefits. Nonetheless, this study provides recommendations for urban planning. However, further research should explore the mechanisms underlying the complex cardiovascular health effects of elderly-supportive infrastructure.

## Conclusions

In this study, results suggested that a high level of elderly-supportive infrastructure may offset the adverse effects of short-term PM_2.5_ exposure on CVD hospitalisations, particularly for stroke and IHD. However, further research should verify its long-term effects and underlying mechanisms. These findings provide valuable insights for cities undergoing urbanisation, highlighting the importance of incorporating health considerations, especially cardiovascular and metabolic health, into urban planning. Moreover, environmental planning and construction that protects the cardiovascular health of the elderly is crucial for the well-being of such individuals.

## References

[1] World urbanization prospects - population division - united nations [Internet]. [cited 2023 Nov 17]. Available from: https://population.un.org/wup/

[2] Gao J, O’Neill BC. Mapping global urban land for the 21st century with data-driven simulations and shared socioeconomic pathways. Nat Commun. 2020;11:11. doi: 10.1038/s41467-020-15788-7 32385275 PMC7210308

[3] Tang Y-X, Zhang Y-T, Xu Y-J, et al. Exposure to ambient particulate matter and hyperuricemia: an eight-year prospective cohort study on male traffic officers in China. Ecotoxicol Environ Saf. 2023;249:114354. doi: 10.1016/j.ecoenv.2022.114354 36508833

[4] de Snyder VNS, Friel S, Fotso JC, et al. Social conditions and urban health inequities: realities, challenges and opportunities to transform the urban landscape through research and action. J Urban Health. 2011;88:1183–14. doi: 10.1007/s11524-011-9609-y 21850555 PMC3232417

[5] Veitch J, Biggs N, Deforche B, et al. What do adults want in parks? A qualitative study using walk-along interviews. BMC Public Health. 2022;22:753. doi: 10.1186/s12889-022-13064-5 35421959 PMC9008398

[6] Josey MJ, Moore S. The influence of social networks and the built environment on physical inactivity: a longitudinal study of urban-dwelling adults. Health Place. 2018;54:62–68. doi: 10.1016/j.healthplace.2018.08.016 30245244 PMC6240490

[7] Bower M, Kent J, Patulny R, et al. The impact of the built environment on loneliness: a systematic review and narrative synthesis. Health Place. 2023;79:102962. doi: 10.1016/j.healthplace.2022.102962 36623467

[8] Li G, Fang C, Wang S, Sun S. The effect of economic growth, urbanization, and industrialization on fine particulate matter (PM2.5) concentrations in China. Environ Sci Technol. 2016;50:11452–11459. doi: 10.1021/acs.est.6b02562 27709931

[9] Li M, Li Y, Liu Z, Hystad P, Rangarajan S, Tse LA, et al. Associations of perceived built environment characteristics using NEWS questionnaires with all-cause mortality and major cardiovascular diseases: the prospective urban rural epidemiology (pure)-China study. Environ Int. 2024;187:108627. doi: 10.1016/j.envint.2024.108627 38636273

[10] Bhatnagar A. Environmental determinants of cardiovascular disease. Circ Res. 2017;121:162–180. doi: 10.1161/CIRCRESAHA.117.306458 28684622 PMC5777598

[11] Giurgescu C, Nowak AL, Gillespie S, Nolan TS, Anderson CM, Ford JL, et al. Neighborhood environment and DNA methylation: implications for cardiovascular disease risk. J Urban Health. 2019;96:23–34. doi: 10.1007/s11524-018-00341-1 30635842 PMC6430282

[12] Sallis JF, Floyd MF, Rodríguez DA, Saelens BE. Role of built environments in physical activity, obesity, and cardiovascular disease. Circulation. 2012;125:729–737. doi: 10.1161/CIRCULATIONAHA.110.969022 22311885 PMC3315587

[13] Liu M, Meijer P, Lam TM, Timmermans EJ, Grobbee DE, Beulens JWJ, et al. The built environment and cardiovascular disease: an umbrella review and meta-meta-analysis. Eur J Prev Cardiol. 2023;30:1801–1827. doi: 10.1093/eurjpc/zwad241 37486178

[14] Yeager RA, Smith TR, Bhatnagar A. Green environments and cardiovascular health. Trends Cardiovasc Med. 2020;30:241–246. doi: 10.1016/j.tcm.2019.06.005 31248691 PMC7995555

[15] Wing JJ, August E, Adar SD, Dannenberg L, Hajat A, Sánchez BN, et al. Change in neighborhood characteristics and change in coronary artery calcium. Circulation. 2016;134:504–513. doi: 10.1161/CIRCULATIONAHA.115.020534 27528645 PMC4991627

[16] Hoffmann B, Moebus S, Mӧhlenkamp S, Stang A, Lehmann N, Dragano N, et al. Residential exposure to traffic is associated with coronary atherosclerosis. Circulation. 2007;116:489–496. doi: 10.1161/CIRCULATIONAHA.107.693622 17638927

[17] Wang Y, Wellenius GA, Hickson DA, Gjelsvik A, Eaton CB, Wyatt SB. Residential proximity to traffic-related pollution and atherosclerosis in 4 vascular beds among African-American adults: results from the jackson heart study. Am J Epidemiol. 2016;184:732–743. doi: 10.1093/aje/kww080 27789446 PMC5141947

[18] Bevan GH, Freedman DA, Lee EK, Rajagopalan S, Al-Kindi SG. Association between ambient air pollution and county-level cardiovascular mortality in the United States by social deprivation index. Am Heart J. 2021;235:125–131. doi: 10.1016/j.ahj.2021.02.005 33592167

[19] Chan OF, Liu Y, Guo Y, Lu S, Chui CHK, Ho HC, et al. Neighborhood built environments and cognition in later life. Aging Ment Health. 2023;27:466–474. doi: 10.1080/13607863.2022.2046697 35285762

[20] Zhou Y, Wu Q, Li C, Ding L. Association between community environment and depressive symptoms among Chinese middle-aged and older adults: evidence from national longitudinal surveys from 2011 to 2018. J Gerontol A Biol Sci Med Sci. 2022;77:2265–2271. 35137075 10.1093/gerona/glac032

[21] Fan VS, Mahadevan R, Leung J. Effect of income inequality, community infrastructure and individual stressors on adult depression. Health Promot Int. 2021;36:46–57. doi: 10.1093/heapro/daaa036 32277828

[22] Wang S, Yung EHK, Cerin E, Yu Y, Yu P. Older People’s usage pattern, satisfaction with community facility and well-being in Urban Old districts. IJERPH. 2022;19:10297. doi: 10.3390/ijerph191610297 36011933 PMC9408318

[23] Urbano T, Chiari A, Malagoli C, Cherubini A, Bedin R, Costanzini S, et al. Particulate matter exposure from motorized traffic and risk of conversion from mild cognitive impairment to dementia: an Italian prospective cohort study. Environ Res. 2023;222:115425. doi: 10.1016/j.envres.2023.115425 36740156

[24] Wilker EH, Martinez-Ramirez S, Kloog I, Schwartz J, Mostofsky E, Koutrakis P, et al. Fine particulate matter, residential proximity to Major roads, and markers of small vessel disease in a memory study population. J Alzheimers Dis. 2016;53:1315–1323. 27372639 10.3233/JAD-151143 PMC4992433

[25] Grazuleviciene R, Dedele A, Danileviciute A, Vencloviene J, Grazulevicius T, Andrusaityte S, et al. The influence of proximity to city parks on blood pressure in early pregnancy. Int J Environ Res Public Health. 2014;11:2958–2972. doi: 10.3390/ijerph110302958 24619158 PMC3987015

[26] Garg G, Tedla YG, Ghosh AS, Mohottige D, Kolak M, Wolf M, et al. Supermarket proximity and risk of hypertension, diabetes, and CKD: a retrospective cohort study. Am J Kidney Dis. 2023;81:168–178. doi: 10.1053/j.ajkd.2022.07.008 36058428 PMC10286129

[27] Michimi A, Wimberly MC. Associations of supermarket accessibility with obesity and fruit and vegetable consumption in the conterminous United States. Int J Health Geogr. 2010;9:49. doi: 10.1186/1476-072X-9-49 20932312 PMC2959055

[28] Zhang K, Lovasi GS, Odden MC, Michael YL, Newman AB, Arnold AM, et al. Association of retail environment and neighborhood socioeconomic status with mortality among community-dwelling older adults in the United States: cardiovascular health study. J Gerontol A Biol Sci Med Sci. 2022;77: 2240–2247. 34669918 10.1093/gerona/glab319 PMC9678200

[29] Aretz B, Costa R, Doblhammer G, Janssen F. The association of unhealthy and healthy food store accessibility with obesity prevalence among adults in the Netherlands: a spatial analysis. SSM Popul Health. 2023;21:101332. doi: 10.1016/j.ssmph.2022.101332 36654966 PMC9841217

[30] Kampa M, Castanas E. Human health effects of air pollution. Environ Pollut. 2008;151:362–367. doi: 10.1016/j.envpol.2007.06.012 17646040

[31] Rahman MM, Alam K. Clean energy, population density, urbanization and environmental pollution nexus: evidence from Bangladesh. Renew Energy. 2021;172:1063–1072. doi: 10.1016/j.renene.2021.03.103

[32] Liu Z, Fang C, Sun B, Liao X. Governance matters: urban expansion, environmental regulation, and PM2.5 pollution. Sci Total Environ. 2023;876:162788. doi: 10.1016/j.scitotenv.2023.162788 36907424

[33] Wang M, Zhou T, Song Y, Li X, Ma H, Hu Y, et al. Joint exposure to various ambient air pollutants and incident heart failure: a prospective analysis in UK Biobank. Eur Heart J. 2021;42:1582–1591. doi: 10.1093/eurheartj/ehaa1031 33527989 PMC8060055

[34] Seposo X, Ueda K, Sugata S, Yoshino A, Takami A. Short-term effects of air pollution on daily single- and co-morbidity cardiorespiratory outpatient visits. Sci Total Environ. 2020;729:138934. doi: 10.1016/j.scitotenv.2020.138934 32371210

[35] Hu X, Nie Z, Ou Y, Lin L, Qian Z, Vaughn MG, et al. Long-term exposure to ambient air pollution, circadian syndrome and cardiovascular disease: a nationwide study in China. Sci Total Environ. 2023;868:161696. doi: 10.1016/j.scitotenv.2023.161696 36682545

[36] Raza A, Bellander T, Bero-Bedada G, Dahlquist M, Hollenberg J, Jonsson M, et al. Short-term effects of air pollution on out-of-hospital cardiac arrest in Stockholm. Eur Heart J. 2014;35:861–868. doi: 10.1093/eurheartj/eht489 24302272

[37] Cho E, Kang Y, Cho Y. Effects of fine particulate matter on cardiovascular disease morbidity: a study on seven metropolitan cities in South Korea. Int J Public Health. 2022;67:1604389. doi: 10.3389/ijph.2022.1604389 35652123 PMC9149776

[38] deSouza P, Braun D, Parks RM, Schwartz J, Dominici F, Kioumourtzoglou M-A. Nationwide study of short-term exposure to fine particulate matter and cardiovascular hospitalizations among medicaid enrollees. Epidemiology. 2021;32:6–13. doi: 10.1097/EDE.0000000000001265 33009251 PMC7896354

[39] Cudjoe TKM, Roth DL, Szanton SL, Wolff JL, Boyd CM, Thorpe RJ, et al. The epidemiology of social isolation: national health and aging trends study. J Gerontol B Psychol Sci Soc Sci. 2020;75:107–113. doi: 10.1093/geronb/gby037 29590462 PMC7179802

[40] Winters M, Buehler R, Götschi T. Policies to promote active travel: evidence from reviews of the literature. Curr Environ Health Rep. 2017;4:278–285. doi: 10.1007/s40572-017-0148-x 28695486

[41] Xie H, Wang X, Wang Z, Shi Z, Hu X, Lin H, et al. Mismatch between infrastructure supply and demand within a 15-minute living circle evaluation in Fuzhou, China. Heliyon. 2023;9:e20130. doi: 10.1016/j.heliyon.2023.e20130 37809587 PMC10559911

[42] Jeste DV, Blazer DG, Buckwalter KC, Cassidy K-LK, Fishman L, Gwyther LP, et al. Age-friendly communities initiative: public health approach to promoting successful aging. Am J Geriatr Psychiatry. 2016;24:1158–1170. doi: 10.1016/j.jagp.2016.07.021 27742528

[43] Xie B, Zheng Y, Li Z, An Z. Influence of urban high-density living environment on stroke risk: a case study of Wuhan. City Plann Rev. 2021;45:30–39.

[44] Wuhan Municipal Bureau of Statistics [Internet]. [cited 2024 Jun 10]. Available from: https://tjj.wuhan.gov.cn/tjfw/tjgb/202404/t20240405_2384677.shtml

[45] National Bureau of Statistics of China [Internet]. [cited 2024 Dec 20]. Available from: https://www.stats.gov.cn/english/

[46] Ageing and health [Internet]. [cited 2024 Dec 20]. Available from: https://www.who.int/news-room/fact-sheets/detail/ageing-and-health

[47] Free 2025 ICD-10-CM codes [internet]. [cited 2024 Dec 20]. Available from: https://www.icd10data.com/ICD10CM/Codes

[48] Wei J, Li Z. ChinaHighPM2.5: big data seamless 1 km ground-level PM2.5 dataset for China [internet]. Zenodo. 2019 [cited 2024 Jun 25]. Available from: https://zenodo.org/records/6398971

[49] Wei J, Li Z, Cribb M, Huang W, Xue W, Sun L, et al. Improved 1 km resolution PM _2.5_ estimates across China using enhanced space–time extremely randomized trees. Atmos Chem Phys. 2020;20:3273–3289. doi: 10.5194/acp-20-3273-2020

[50] Wei J, Li Z, Lyapustin A, Sun L, Peng Y, Xue W, et al. Reconstructing 1-km-resolution high-quality PM2.5 data records from 2000 to 2018 in China: spatiotemporal variations and policy implications. Remote Sens Of Environ. 2021;252:112136. doi: 10.1016/j.rse.2020.112136

[51] Agrometeorological indicators from 1979 to present derived from reanalysis [Internet]. [cited 2024 Apr 25]. Available from: https://cds.climate.copernicus.eu/cdsapp#!/dataset/10.24381/cds.6c68c9bb?tab=overview

[52] Timmis A, Vardas P, Townsend N, Torbica A, Katus H, De Smedt D, et al. European society of cardiology: cardiovascular disease statistics 2021. Eur Heart J. 2022;43:716–799. doi: 10.1093/eurheartj/ehab892 35016208

[53] Yang H, Li S, Sun L, Zhang X, Cao Z, Xu C, et al. Smog and risk of overall and type-specific cardiovascular diseases: a pooled analysis of 53 cohort studies with 21.09 million participants. Environ Res. 2019;172:375–383. doi: 10.1016/j.envres.2019.01.040 30825688

[54] Feng S, Gao D, Liao F, Zhou F, Wang X. The health effects of ambient PM2.5 and potential mechanisms. Ecotoxicol Environ Saf. 2016;128:67–74. doi: 10.1016/j.ecoenv.2016.01.030 26896893

[55] Münzel T, Gori T, Al-Kindi S, Deanfield J, Lelieveld J, Daiber A, et al. Effects of gaseous and solid constituents of air pollution on endothelial function. Eur Heart J. 2018;39:3543–3550. doi: 10.1093/eurheartj/ehy481 30124840 PMC6174028

[56] Tanaka M, Okuda T, Itoh K, Ishihara N, Oguro A, Fujii-Kuriyama Y, et al. Polycyclic aromatic hydrocarbons in urban particle matter exacerbate movement disorder after ischemic stroke via potentiation of neuroinflammation. Part Fibre Toxicol. 2023;20:6. doi: 10.1186/s12989-023-00517-x 36797786 PMC9933276

[57] Ying Z, Xu X, Bai Y, Zhong J, Chen M, Liang Y, et al. Long-term exposure to concentrated ambient PM2.5 increases mouse blood pressure through abnormal activation of the sympathetic nervous system: a role for hypothalamic inflammation. Environ Health Perspect. 2014;122:79–86. doi: 10.1289/ehp.1307151 24240275 PMC3888575

[58] Lederer AM, Fredriksen PM, Nkeh-Chungag BN, Everson F, Strijdom H, De Boever P, et al. Cardiovascular effects of air pollution: current evidence from animal and human studies. Am J Physiol-Heart Circulatory Physiol. 2021;320:H1417–H1439. doi: 10.1152/ajpheart.00706.2020 33513082

[59] Ma M, Shi L, Xie W, Zhu Q, Luo J, Liao S, et al. Coupling coordination degree of healthcare resource supply, demand and elderly population change in China. Int J Equity Health. 2024;23:147. doi: 10.1186/s12939-024-02236-x 39049064 PMC11270932

[60] Perry AS, Dooley EE, Master H, Spartano NL, Brittain EL, Pettee Gabriel K. Physical activity over the lifecourse and cardiovascular disease. Circ Res. 2023;132:1725–1740. 37289900 10.1161/CIRCRESAHA.123.322121 PMC10254078

[61] Zhang Y-B, Chen C, Pan X-F, Guo J, Li Y, Franco OH, et al. Associations of healthy lifestyle and socioeconomic status with mortality and incident cardiovascular disease: two prospective cohort studies. BMJ. 2021;373:n604. doi: 10.1136/bmj.n604 33853828 PMC8044922

[62] Christiansen J, Lund R, Qualter P, Andersen CM, Pedersen SS, Lasgaard M. Loneliness, social isolation, and chronic disease outcomes. Annals Behavioral Medicine. 2021;55:203–215. doi: 10.1093/abm/kaaa044 32865550

[63] Stamatakis E, Gale J, Bauman A, Ekelund U, Hamer M, Ding D. Sitting time, physical activity, and risk of mortality in adults. J Am Coll Cardiol. 2019;73:2062–2072. doi: 10.1016/j.jacc.2019.02.031 31023430

[64] Stappers NEH, Bekker MPM, Jansen MWJ, Kremers SPJ, De Vries NK, Schipperijn J, et al. Effects of major urban redesign on sedentary behavior, physical activity, active transport and health-related quality of life in adults. BMC Public Health. 2023;23:1157. doi: 10.1186/s12889-023-16035-6 37322454 PMC10267553

[65] Mason KE, Pearce N, Cummins S. Do neighbourhood characteristics act together to influence BMI? A cross-sectional study of urban parks and takeaway/fast-food stores as modifiers of the effect of physical activity facilities. Soc Sci Med. 2020;261:113242. doi: 10.1016/j.socscimed.2020.113242 32745823

[66] Soma Y, Sato A, Tsunoda K, Kitano N, Jindo T, Abe T, et al. Relationships between participation in volunteer-managed exercises, distance to exercise facilities, and interpersonal social networks in older adults: a cross-sectional study in Japan. IJERPH. 2021;18:11944. doi: 10.3390/ijerph182211944 34831701 PMC8623852

[67] Jiang J, Xia Z, Sun X, Wang X, Luo S. Social infrastructure and street networks as critical infrastructure for aging friendly community design: mediating the effect of physical activity. IJERPH. 2022;19:11842. doi: 10.3390/ijerph191911842 36231144 PMC9565500

[68] Xiao Y, Chen S, Miao S, Yu Y. Exploring the mediating effect of physical activities on built environment and obesity for elderly people: evidence from Shanghai, China. Front Public Health. 2022;10:853292. doi: 10.3389/fpubh.2022.853292 35359789 PMC8961803

[69] Chen S, Wang T, Bao Z, Lou V. A path analysis of the effect of neighborhood built environment on public health of older adults: a Hong Kong study. Front Public Health. 2022;10:861836. doi: 10.3389/fpubh.2022.861836 35359794 PMC8964032

[70] Imazio M, Mardigyan V, Andreis A, Franchin L, De Biasio M, Collini V. New developments in the management of recurrent pericarditis. Can J Cardiol. 2023;39:1103–1110. doi: 10.1016/j.cjca.2023.04.008 37075863

[71] Werdan K, Dietz S, Löffler B, Niemann S, Bushnaq H, Silber R-E, et al. Mechanisms of infective endocarditis: pathogen–host interaction and risk states. Nat Rev Cardiol. 2014;11:35–50. doi: 10.1038/nrcardio.2013.174 24247105

[72] Moncla L-H, Briend M, Bossé Y, Mathieu P. Calcific aortic valve disease: mechanisms, prevention and treatment. Nat Rev Cardiol. 2023;20:546–559. doi: 10.1038/s41569-023-00845-7 36829083

[73] Dillmann WH. Diabetic cardiomyopathy. Circ Res. 2019;124:1160–1162. doi: 10.1161/CIRCRESAHA.118.314665 30973809 PMC6578576

[74] Avery CL, Mills KT, Williams R, McGraw KA, Poole C, Smith RL, et al. Estimating error in using residential outdoor PM2.5 concentrations as proxies for personal exposures: a meta-analysis. Environ Health Perspect. 2010;118:673–678. doi: 10.1289/ehp.0901158 20075021 PMC2866684

